# Supplementary material for: Tracing cancer evolution and heterogeneity using Hi-C
Source: Nat Commun. 2023 Nov 6;14:7111. doi: 10.1038/s41467-023-42651-2 (PMC10628133; doi:10.1038/s41467-023-42651-2)
Supplement: Supplementary file 3 — Reporting Summary [file 41467_2023_42651_MOESM3_ESM.pdf]

nature portfolio

Corresponding author(s): Yun S. Song
Daniel S. Rokhsar
Dirk Hockemeyer

Last updated by author(s): Sep 3, 2023

Reporting Summary

Nature Portfolio wishes to improve the reproducibility of the work that we publish. This form provides structure for consistency and transparency in reporting. For further information on Nature Portfolio policies, see our Editorial Policies and the Editorial Policy Checklist.

Statistics

For all statistical analyses, confirm that the following items are present in the figure legend, table legend, main text, or Methods section.

n/a	Confirmed	
☐	☒	The exact sample size (*n*) for each experimental group/condition, given as a discrete number and unit of measurement
☒	☐	A statement on whether measurements were taken from distinct samples or whether the same sample was measured repeatedly
☒	☐	The statistical test(s) used AND whether they are one- or two-sided<br>*Only common tests should be described solely by name; describe more complex techniques in the Methods section.*
☐	☒	A description of all covariates tested
☐	☒	A description of any assumptions or corrections, such as tests of normality and adjustment for multiple comparisons
☐	☒	A full description of the statistical parameters including central tendency (e.g. means) or other basic estimates (e.g. regression coefficient) AND variation (e.g. standard deviation) or associated estimates of uncertainty (e.g. confidence intervals)
☒	☐	For null hypothesis testing, the test statistic (e.g. *F*, *t*, *r*) with confidence intervals, effect sizes, degrees of freedom and *P* value noted<br>*Give P values as exact values whenever suitable.*
☐	☒	For Bayesian analysis, information on the choice of priors and Markov chain Monte Carlo settings
☐	☒	For hierarchical and complex designs, identification of the appropriate level for tests and full reporting of outcomes
☒	☐	Estimates of effect sizes (e.g. Cohen's *d*, Pearson's *r*), indicating how they were calculated

Our web collection on statistics for biologists contains articles on many of the points above.

Software and code

Policy information about availability of computer code

Data collection	Hi-C data for GM12878 was downloaded from the 4D Nucleome website: https://data.4dnucleome.org/files-processed/4DNFI1UEG1HD/
Data analysis	Code for HiDENSEC accompanying this paper can be accessed at: https://github.com/songlab-cal/HiDENSEC<br>HiNT was run with version: 2.2.7.<br>hic_breakfinder38 was downloaded from https://github.com/dixonlab/hic_breakfinder, using commit 30a0dcc6d01859797d7c263df7335fd2f52df7b8 (last updated in 2018).<br>EagleC was downloaded from https://github.com/XiaoTaoWang/EagleC.

For manuscripts utilizing custom algorithms or software that are central to the research but not yet described in published literature, software must be made available to editors and reviewers. We strongly encourage code deposition in a community repository (e.g. GitHub). See the Nature Portfolio guidelines for submitting code & software for further information.

Data

Policy information about availability of data

All manuscripts must include a data availability statement. This statement should provide the following information, where applicable:

- Accession codes, unique identifiers, or web links for publicly available datasets
- A description of any restrictions on data availability
- For clinical datasets or third party data, please ensure that the statement adheres to our policy

The Hi-C and capture sequencing (UCSF500 or Exome sequencing) data generated in this study have been deposited in the dbGap database under accession code phs001550.v3.p1 [https://www.ncbi.nlm.nih.gov/projects/gap/cgi-bin/study.cgi?study_id=phs001550.v3.p1]. Additionally, the Hi-C data generated in this study for the in vitro mixtures have been deposited in the SRA database under accession code PRJNA849975 [https://www.ncbi.nlm.nih.gov/sra/PRJNA849975]. Source data for figures is available on Zenodo [https://doi.org/10.5281/zenodo.8313343].

Human research participants

Policy information about studies involving human research participants and Sex and Gender in Research.

Reporting on sex and gender	Patient-1 is Male and was born in 1967. Patient-2 is Female and was born in 1956. Patient-3 is Female and was born in 1915. Patient-4 is Female and was born in 1956.
Population characteristics	N/A
Recruitment	N/A
Ethics oversight	Archival formalin-fixed, paraffin-embedded (FFPE) melanoma samples were retrieved from the archives of the University of California San Francisco Dermatopathology service, under an IRB approved protocol (11-07951) that granted us a waiver of consent to work with leftover archival tissue. No participant was paid.

Note that full information on the approval of the study protocol must also be provided in the manuscript.

Field-specific reporting

Please select the one below that is the best fit for your research. If you are not sure, read the appropriate sections before making your selection.

☒ Life sciences ☐ Behavioural & social sciences ☐ Ecological, evolutionary & environmental sciences

For a reference copy of the document with all sections, see nature.com/documents/nr-reporting-summary-flat.pdf

Life sciences study design

All studies must disclose on these points even when the disclosure is negative.

Sample size	Samples were selected based on their histological assessment and no statistical methods were used to determine sample size.
Data exclusions	Only samples that yielded Hi-C data were included.
Replication	Orthogonal capture sequencing techniques and prior sequencing data were used to validate the results outlined in the paper.
Randomization	Randomization was not used in the experimental design.
Blinding	Samples were not blinded.

Reporting for specific materials, systems and methods

We require information from authors about some types of materials, experimental systems and methods used in many studies. Here, indicate whether each material, system or method listed is relevant to your study. If you are not sure if a list item applies to your research, read the appropriate section before selecting a response.

Materials & experimental systems

n/a	Involved in the study
☒	☐ Antibodies
☐	☒ Eukaryotic cell lines
☒	☐ Palaeontology and archaeology
☒	☐ Animals and other organisms
☒	☐ Clinical data
☒	☐ Dual use research of concern

Methods

n/a	Involved in the study
☒	☐ ChIP-seq
☒	☐ Flow cytometry
☒	☐ MRI-based neuroimaging

Eukaryotic cell lines

Policy information about cell lines and Sex and Gender in Research

Cell line source(s)	HCC1187C is available through the American tissue culture collection.
Authentication	Genotyping was performed to authenticate the cell line.
Mycoplasma contamination	Cell lines were tested negative for mycoplasma.
Commonly misidentified lines (See ICLAC register)	N/A
