# Supplementary material for: Tracing cancer evolution and heterogeneity using Hi-C
Source: Nat Commun. 2023 Nov 6;14:7111. doi: 10.1038/s41467-023-42651-2 (PMC10628133; doi:10.1038/s41467-023-42651-2)
Supplement: Supplementary file 4 — Description of Additional Supplementary Files [file 41467_2023_42651_MOESM4_ESM.pdf]

Description of Additional Supplementary Files

File Name: Supplementary Data 1
Description: *in vitro* mixtures of human and mouse cells show negligible ligation across species

File Name: Supplementary Data 2
Description: Hi-C sequencing statistics for all samples

File Name: Supplementary Data 3
Description: 50 Kb resolution interchromosomal translocation locations identified manually in Juicebox for all samples

File Name: Supplementary Data 4
Description: Tumor purity estimates for Patient - 1 and Patient - 2

File Name: Supplementary Data 5
Description: Fraction of cells belonging to various cell types in samples of Patient - 3
